# Physical activity across the curriculum: year one process evaluation results

**DOI:** 10.1186/1479-5868-5-36

**Published:** 2008-07-07

**Authors:** Cheryl A Gibson, Bryan K Smith, Katrina D DuBose, J Leon Greene, Bruce W Bailey, Shannon L Williams, Joseph J Ryan, Kristin H Schmelzle, Richard A Washburn, Debra K Sullivan, Matthew S Mayo, Joseph E Donnelly

**Affiliations:** 1 Department of Internal Medicine, University of Kansas School of Medicine, 3901 Rainbow Blvd., Mail Stop 1020, Kansas City, KS, 66160, USA; 2 The Center for Physical Activity and Weight Management, The Schiefelbusch Institute for Lifespan Studies, 1301 Sunnyside Ave, Robinson Center Rm 100, The University of Kansas, Lawrence, KS, 66045, USA; 3 Department of Exercise & Sport Science, 174 Minges Coliseum, East Carolina University, Greenville, NC, 27858, USA; 4 Department of Health, Sport, and Exercise Sciences, 1301 Sunnyside Ave, Robinson Center Room 161, The University of Kansas, Lawrence, KS, 66045, USA; 5 Department of Exercise and Health Sciences, University of Massachusetts Boston, 100 Morrissey Blvd., Boston, MA, 02125-3393, USA; 6 International Life Sciences Institute, Center for Health Promotion, Physical Activity and Nutrition (PAN) Program, 2295 Parklake Drive, Suite 450, Atlanta, GA, 30345, USA; 7 Department of Psychology, University of Central Missouri, Warrensburg, MO, 64093, USA; 8 Center for Biostatistics and Advanced Informatics, University of Kansas School of Medicine, 3901 Rainbow Blvd, Mail Stop 1026, Kansas City, KS, 66160, USA; 9 Department of Dietetics and Nutrition, University of Kansas School of Allied Health, 3901 Rainbow Blvd, Mail Stop 4013, Kansas City, KS, 66160, USA; 10 Department of Biostatistics, University of Kansas School of Medicine, 3901 Rainbow Blvd, Mail Stop 1026, Kansas City, KS, 66160, USA

## Abstract

**Background:**

Physical Activity Across the Curriculum (PAAC) is a 3-year elementary school-based intervention to determine if increased amounts of moderate intensity physical activity performed in the classroom will diminish gains in body mass index (BMI). It is a cluster-randomized, controlled trial, involving 4905 children (2505 intervention, 2400 control).

**Methods:**

We collected both qualitative and quantitative process evaluation data from 24 schools (14 intervention and 10 control), which included tracking teacher training issues, challenges and barriers to effective implementation of PAAC lessons, initial and continual use of program specified activities, and potential competing factors, which might contaminate or lessen program effects.

**Results:**

Overall teacher attendance at training sessions showed exceptional reach. Teachers incorporated active lessons on most days, resulting in significantly greater student physical activity levels compared to controls (p < 0.0001). Enjoyment ratings for classroom-based lessons were also higher for intervention students. Competing factors, which might influence program results, were not carried out at intervention or control schools or were judged to be minimal.

**Conclusion:**

In the first year of the PAAC intervention, process evaluation results were instrumental in identifying successes and challenges faced by teachers when trying to modify existing academic lessons to incorporate physical activity.

## Background

In the United States, the prevalence of overweight among children increased dramatically between 1986 and 1998 [1], while the most recent estimates from the National Health and Nutrition Examination Survey (NHANES, 1999–2002) indicate that the high levels of overweight among children have not changed [2]. Therefore, decreasing the prevalence of obesity in children remains a public health priority. The US Department of Health and Human Services, through its many agencies, is leading a new effort to promote an initiative to improve the health of all Americans–Steps to a HealthierUS [3]. One component of the initiative addresses the potential of school-based programs to help young people adopt and maintain healthy eating and physical activity behaviors to prevent and control obesity and other chronic diseases.

Public schools are an ideal site for interventions designed for the primary prevention of obesity in children. According to the most recent enrollment figures, 98% of children were enrolled in regular schools, representing more than 47 million students in the United States[4]. Additionally, required attendance along with zero tolerance policies for truancy provide health promoters access to numerous children thus enabling repeated exposure to intervention activities [5]. School policies can be modified and teachers can be trained in new methods of service delivery. As a result, schools can easily provide a mechanism for maintenance and dissemination so that successful interventions may continue after the initial intervention phase, and may be dispersed throughout school systems.

Ironically, schools also are a barrier to physical activity. Children are required to sit quietly for the majority of the day to receive academic instruction. A typical school day can be best described as sedentary. Historically, schools have provided physical activity for elementary school children through free play or recess, and through organized physical education classes. However, time allocated for recess has declined or has been eliminated to provide additional time for academic instruction [6]. Further, results from the School Health Policies and Programs Study, a national survey conducted to assess school health policies and programs, indicate that very few states provide daily physical education classes or its equivalent [5].

In an attempt to diminish childhood obesity, we developed a minimal intervention model to increase physical activity levels, since increased physical activity has shown promise with weight management in children [7,8]. Regular classroom teachers were taught to deliver existing academic lessons using physical activity in an approach called Physical Activity Across the Curriculum (PAAC). We partnered with The International Life Sciences Institute Center for Health Promotion that has developed a similar program called TAKE 10!^® ^It is a classroom-based physical activity program for kindergarten to fifth grade students. TAKE 10!^® ^provides physical activities that are linked to academic objectives and delivered in 10-minute sessions. This article provides a review of the overall design and first-year process evaluation results of PAAC.

Monitoring the delivery of services to evaluate the actual implementation of PAAC was undertaken for a number of purposes. A large proportion of programs that fail to show positive results are really failures to deliver the interventions as designed [9]. The process evaluation was specifically designed to monitor the delivery of PAAC lessons and to identify faults and deficiencies so that implementation failure or dilution of treatment could be avoided. For example, a lack of commitment on the part of teachers might result in minimal delivery of physically active lessons or "ritual compliance" [10].

### Physical activity across the curriculum: intervention and research design

PAAC is a cluster-randomized controlled, elementary school-based trial, involving 4905 children (2505 intervention, 2400 control) in 24 schools (14 intervention and 10 control). The study is being carried out in public elementary schools located in the Midwest region of the United States. The primary aim of the intervention is to determine if 90 minutes of moderate intensity physical activity delivered as part of academic instruction will diminish gains in obesity as measured by body mass index (BMI). Secondary outcomes are assessed in a sub-sample of children to provide additional means of evaluating the impact of PAAC, including measures of metabolic fitness, daily physical activity, and academic achievement.

The intervention will be delivered across 3 years in elementary school children in grades 2 through 5. Physical activity is accumulated throughout the school day using regular, existing academic lessons taught in a physically active manner. Lessons link physical activity with specific academic learning objectives for language arts, science, mathematics, and social studies.

#### PAAC background

The International Life Sciences Institute Center for Health Promotion (ILSI CHP) developed the TAKE10!^® ^program to provide children with a brief, classroom-based activity segment one or more times each school day, integrating physical activity with academic content related to health and movement concepts. For this intervention, we joined with ILSI CHP, providing teachers with TAKE10!^® ^material kits that included lesson ideas and worksheets targeted for the appropriate grade level, colorful posters for each classroom, and stickers that can be used to chart class progress on TAKE10!^® ^lessons. For PAAC, we developed and refined classroom-based lesson plans to be delivered through physical activity. All physically active lesson plans were designed to be consistent with the cognitive and motor development of students at each grade level. In addition, we designed examples of physically active lessons to be taught as part of the regular school curriculum, integrated within mathematics, science, language arts, and social studies and delivered by regular classroom teachers. Sample lessons were included in a 3-ring notebook and provided to classroom teachers as a resource. Lesson examples were described in a cookbook format that allowed teachers to identify the concept underlying the suggested physical activity, specific objectives, and materials needed.

### Process evaluation methods

We used evaluation components as described and defined by Linnan and Steckler [11] and Baranowski and Stables [12] to provide an overall framework for our key process evaluation measures. The primary aims of the process evaluation were to monitor the extent to which the teachers delivered PAAC as originally planned (i.e., fidelity), to track the extent to which the intervention had been implemented and received by the teachers (i.e., implementation and reach), and to assess the extent to which the teachers and students actively engaged in PAAC lessons across the school year (i.e., dose received, initial and continual use of PAAC activities). Another aim of the evaluation was to provide a general description of the context in which the PAAC program was conducted. Table 1 summarizes the process components, data sources, data collection instruments, and frequency of measurement. In the following paragraphs, we describe each of the process measures, and the sources and frequency of data collection. The Institutional Review Board of the University of Kansas approved the present study.

**Table 1:** Process evaluation measures

**Evaluation Components**[11,12]	**Data Collection** **Instruments**	**Source**	**Frequency of ** **Measurement**	**Schools**
Reach	Training Attendance Record	Teachers	Annual workshop; as needed when new faculty hired	I
Dose Received	Training Evaluation Survey	Teachers	Annual workshop; as needed when new faculty hired	I
Precision, Accuracy	Anthropometric Reliability Testing	Research Assistants	Quarterly	
Fidelity, Dose received	System for Observing Fitness Instruction Time (SOFIT)	Students	Weekly	I, C
Fidelity	Student Enjoyment	Teachers	End of school year (teachers), weekly (direct observation)	I, C^1^
Fidelity, Dose, Dose received	On-line Weekly Classroom Teacher Survey (Form 1)	Teachers	Weekly	I
Fidelity, Implementation	On-line Classroom Teacher Questionnaire (Form 2)	Teachers	End of school year	I
Fidelity, Exposure	On-line 9-item Classroom Teacher Questionnaire (Form 3)	Teachers	End of school year	I
Reach	Focus Group Attendance Log	Teachers	End of school year	I
Context, Contamination	5-item Competing Factors Survey	Principals	End of school year	I, C

^1 ^Enjoyment of students was rated weekly by research assistants in both intervention and control schools. In addition, enjoyment level of students was reported by intervention teachers only at the end of the school year. I = intervention schools; C = control schools

#### Teacher training

We arranged one-day workshops (approximately 6 hours) for intervention schoolteachers who taught second through fifth grade, using the traditional in-service training mechanism. Schools or individual teachers, depending on policy, received payment for attending the workshop. An elementary physical education specialist led the sessions. Attendance at the workshop training session was recorded as a measure of reach or the extent to which the teachers received training [13], and for those teachers who were unable to attend, training was held on an individual basis or conducted in small groups by geographic location of schools.

At the end of the workshop, teachers completed a 15-item evaluation survey. In addition to demographic information, several items on the survey asked teachers to rate how well organized, appropriate, and relevant the workshop was to their individual classrooms. To gain ideas about enhancing future workshop sessions, we asked teachers to provide advice on how to improve our training sessions and recommendations for additional topics to be covered at future workshops.

#### Quality assurance

In order to assure precision and accuracy of process evaluation and outcome measurements, rigorous quality control procedures were employed. All research assistants participated in several training sessions prior to the initiation of data collection. During these training sessions, research assistants reviewed and practiced standardized protocols for height, weight, and circumference measurements. Following training, all research assistants completed reliability testing. Research assistants were also trained to make direct observations of physical activity levels within the classroom and learned how to record them uniformly. Using a time-moment sampling technique called the System for Observing Fitness Instruction Time (SOFIT) [14], research assistants practiced recording the physical activity intensity level of students in several classrooms, using a Likert-type scale (e.g. 1 = lying down, 2 = sitting, 3 = standing, 4 = walking, 5 = jogging/running). Ratings were compared across research assistants for reliability and percentage agreement was calculated. To control drift across time, reliability testing was conducted on quarterly intervals.

In addition to standardized protocols and reliability testing, investigators and research staff met weekly to discuss issues related to program implementation and monitoring. During the meetings, the study coordinator shared the results of the weekly teacher evaluations and on-site visits so that the investigators could keep apprised of developments affecting PAAC and gain up-to-date information on how many minutes of PAAC activities were performed by individual teachers and their students. With this information, teachers who were not conducting active lessons at the appropriate levels of intensity or amount of time could be identified quickly and PAAC staff could provide on-site follow-up assistance and support in a timely manner. We targeted moderate intensity activity levels during classroom activities, aiming for students to participate in at least two PAAC lessons for 10 or minutes each day.

#### Direct observation of PAAC lessons

Following completion of baseline assessments, direct observations of individual classrooms were initiated. This timeframe allowed teachers several weeks to incorporate physical activity into their regular lessons with the help of PAAC staff and to gain a sense of mastery with presenting active lessons.

We developed a sampling approach that randomly determined the school to be observed and the day of the week to make observations to determine the activity level of the students as measured by SOFIT ratings. The random sampling approach used a 3:1 ratio for intervention and control schools (i.e., for every three intervention schools selected for direct observation only one control school was visited).

Three students from each classroom were randomly selected to be observed in both control and intervention schools to provide a measure of how active students were during classroom lessons. In 20-second intervals, for up to 10 minutes, the students' physical activity intensity levels were estimated and recorded using the SOFIT rating scheme (i.e., 1 = lying down to 5 = jogging or running). During observations, research assistants also indicated how often teachers participated in active lessons with their students by rating them on a 3-point scale (i.e., none, somewhat, very active participation). This particular measure provided an indication of effective role modeling for active lessons. In addition, research assistants rated overall student enjoyment level during both PAAC lessons and regular classroom lessons at intervention and control schools, using a 5-point scale (1 = not very enjoyable to 5 = very enjoyable). All direct observation ratings for classroom teachers as well as enjoyment of lessons were completed in the same manner at both intervention and control schools.

#### Online teacher self-report questionnaires

On a weekly basis, all intervention teachers were required to log onto a password protected website in order to complete a brief online survey. To complete the survey, teachers indicated in what academic subject(s) physical activity was incorporated and how often they included physical activity into lesson plans for approximately 10 or more minutes. They also reported the number of minutes per day they were using PAAC and estimated the intensity level at which they believed the children were performing (i.e., light, moderate or high intensity levels) so that we could gauge the extent to which the PAAC program was being implemented as designed. If teachers failed to complete their weekly survey, individual teachers were sent an email reminder or were given a hard copy of the evaluation to complete the following week.

At the end of the school year, teachers completed two evaluation forms, which were adapted from the Child and Adolescent Trial for Cardiovascular Health (CATCH) [15-17]. The first survey gathered information about the teacher's level of confidence to instruct and demonstrate to their students how to become more physically active, using a 5-point Likert-type scale (1 = not all confident to 5 = extremely confident). We also asked teachers to rate their level of confidence to incorporate physical activity into lesson plans. In addition to measures of self-efficacy, we asked teachers to indicate how important it is to encourage elementary school children to become more physically active, using a 5-point scale (1 = not at all important to 5 = extremely important). We also asked teachers to rate the level of support for PAAC from other elementary teachers, parents, and the school administration, using a 5-point scale (1 = not at all supportive to 5 = extremely supportive).

In order to gain additional information that would be helpful in guiding the overall design and implementation of PAAC for the following school year, we asked teachers to complete a 9-item online survey, which was adapted from the CATCH Physical Education Observation Form [18]. As part of a regular school day, teachers were asked how often they: 1) increased physical activity by incorporating it into lesson plans; 2) observed children enjoying classroom activities that incorporated physical activity; 3) used physical activity as part of the lesson plans to break the monotony of certain subjects; 4) employed physically active lessons to address various learning styles; 5) enjoyed becoming physically active through implementing movement into lesson plans; 6) enhanced their own sense of well-being on the days that they incorporated physical activity into the curriculum; and, 7) participated in PAAC lessons, which contributed to better classroom management. We also asked how often teachers used the PAAC^® ^notebook as a resource for active lessons as one measure of exposure [12]. Response format included five different options (i.e., not at all, at least once per week, 2 or more times per week, on most days and everyday) from which to choose. Lastly, we asked teachers to report what would help them to increase the number of lesson plans that incorporate physical activity.

#### Focus group discussions

Before the school year ended, we invited teachers to participate in focus group discussions. We held six sessions with approximately 10 to 15 teachers attending each discussion. A moderator's guide [19] was developed to learn more about teacher's perceptions about PAAC (i.e., best and worst features) and the challenges and barriers to achieving 90 minutes of active lessons per week. We also inquired about how we could make the delivery of PAAC lessons easier to adapt to a regular lesson. In addition, we solicited information about potential benefits, both physically and academically, to students and teachers who actively participated in PAAC lessons. We provided a monetary incentive to teachers for their participation. General questions were raised and probes were used to elicit further discussion. At each session, discussions lasted approximately 60 minutes. In addition to note taking by the moderator and assistant moderator, discussions were tape-recorded with permission of the teachers and later transcribed. We examined the transcribed tapes and notes for common themes, employing the content analysis techniques recommended by Miles and colleague [20]. Confirmation of common themes was accomplished by having team members identify a pattern of responses for each of the planned questions separately and then responses were compared as a group to identify common threads that extended throughout the six focus group discussions.

#### Principal surveys

At the end of the school year, principals from both intervention and control schools completed a 5-item survey to help us determine if external or competing factors might have influenced PAAC objectives, posing as possible internal validity threats [21]. We asked if the number of times students participate in physical education class had changed during the course of the year. In addition, we inquired about adoption of any new programs or course content that aimed to increase student physical activity intensity levels or altered the caloric intake or nutritional quality of student's diets. Further, we asked if the school had changed any policies that could potentially influence the amount or content of student's beverages or foods (e.g., access to or contents of vending machines available to students, limits placed on the types of snacks served in the classroom, banning certain foods brought from home, etc.).

#### Statistical analysis

Means, standard deviations, and percentages were computed for descriptive data. A mixed linear model analysis was performed to determine differences between intervention and control groups. The model contained group, days since baseline, and group by day interaction as predictor variables and physical activity level as the outcome variable. Teacher was repeated within school. A mixed linear model analysis was used to determine differences between the modeling level of teachers and physical activity intensity levels. This model included teacher participation and days since baseline as predictor variables, with physical activity level as the outcome variable. Again, teacher was repeated within school. This analysis included only intervention schools. All statistical analyses were conducted using SAS version 9.1 (Research Institute, Cary, NC). The alpha level was set at 0.05 for all analyses.

## Results

### Teacher training

Each workshop training session was well attended with an overall attendance rate of 81% of 2^nd ^through 5^th ^grade teachers (106 out of 135) with 100% of the cohort teachers (i.e., 2^nd ^and 3^rd ^grade teachers) trained. Many resource staff (n = 38) participated in the workshop session in addition to faculty members. Other resource staff included teachers from music, special education, language, and reading, and school principals and graduate student teachers. As noted earlier, for those teachers who were unable to attend the workshop, training was held on an individual basis or conducted in small groups.

The results of the workshop evaluation survey indicated that the majority of attendees felt that the workshop training was well organized, appropriate in depth and scope, relevant to their classroom experience and addressed their specific questions about PAAC. Most of the attendees also reported program materials were clear and appropriate for their classrooms, and the training received would be very helpful when incorporating physical activity into their lesson plans. Additionally, more than 90% of attendees specified that they would recommend the workshop training to other elementary school teachers.

### Direct observation of PAAC/TAKE 10!^® ^lessons

On a weekly basis, observers made contact with 85% (115 out of 135) of the teachers and observed 70% of the classrooms. On average, PAAC staff conducted SOFIT observations on 260 students each week at intervention schools to determine student activity intensity levels. For control schools, PAAC staff observed approximately 89% of the classrooms and measured physical activity intensity levels using SOFIT on 75 students each week. Reasons for not making contact with a teacher or observing a classroom included the following: (1) school cancellations or field trips (n = 119); (2) substitute teacher (n = 43); (3) standardized testing (n = 43); or (4) unable to set teacher visitation appointment (n = 39).

Table 2 displays the physical activity intensity levels reported from the SOFIT observations for intervention and control schools for Year 1. SOFIT observations were performed on a total of 4,515 students in the 2^nd ^through 5^th ^grades (intervention schools: 3,465 students; control schools: 1,050 students). Students in the intervention schools performed significantly greater levels of physical activity in the classroom than students in the control schools; higher scores indicate higher activity intensity levels (intervention students 3.40 ± 0.02 vs control students 2.17 ± 0.03, p < 0.0001). Control students were primarily sitting during academic lessons, whereas students in intervention schools were primarily standing during most PAAC lessons.

**Table 2:** Physical activity levels from the SOFIT for grades 2^nd ^– 5^th ^by intervention status.

	School Type		
	Intervention	Control	All
	n = 3429	n = 1047	n = 4476
Physical Activity Variables	M ± SD	M ± SD	M ± SD

Gender			
Male	49.5%	49.9%	49.6%
Female	50.5%	50.1%	50.4%
Observation time (min)	7.91 ± 2.53	9.66 ± 1.14	8.32 ± 2.40
Physical activity level	3.40 ± 0.52*	2.16 ± 0.25	3.11 ± 0.71

*p < 0.05 from mixed linear model.

**Figure 1:**
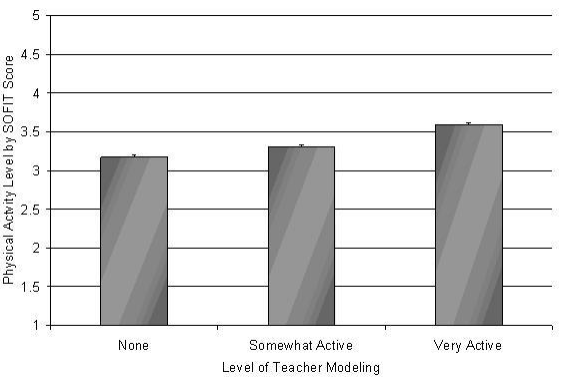
Level of teacher modeling and System for Observing Fitness Instruction Time (SOFIT) score.

During SOFIT observations, staff also indicated the level of participation by teachers during an active lesson. Overall teacher participation was related to SOFIT scores for students in the intervention schools but not the control schools. As shown in Figure 1, as modeling of an active lesson increased by PAAC teachers (i.e., none vs somewhat or very active participation), student SOFIT scores also increased significantly (p < 0.0001). The analysis for the teacher modeling was performed only using the intervention schools because teacher participation had no effect on the activity level of students in the control schools. The SOFIT scores for students in the control schools were less than 2.2 throughout the year regardless of teacher participation.

In addition, ratings of enjoyment of lessons at control schools indicated that most classroom lessons were perceived as neutral (60%). In contrast, the majority of PAAC lessons were rated as somewhat enjoyable (57%) or very enjoyable (36%) while only 6% of lessons were perceived as neutral.

### Online teacher self-report questionnaires

**Figure 2:**
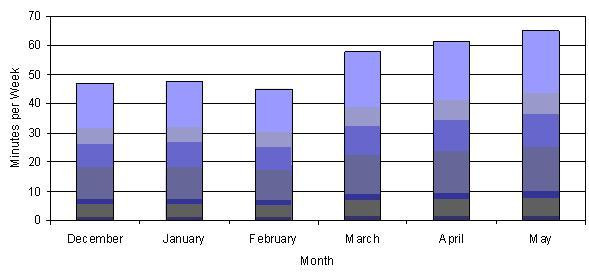
Monthly averages for time spent (minutes/week) incorporating PAAC/Take 10!^® ^as reported by teachers.

Across the school year, approximately 84% of teachers responded to the weekly on-line survey. If a teacher did not respond by the on-line method, hard copies were distributed the following week and research staff entered the collected data. The majority of teachers indicated that they incorporated physical activity into language arts (73%) and math (22%) while other subjects such as science, social studies, art, and music were used much less often. As shown in Figure 2, the number of minutes per week teachers incorporated PAAC into lessons increased considerably over the course of six months, beginning with 47 minutes and ending with 65 minutes per week of active lessons. When estimating the intensity level at which they believed children were performing (i.e., light, moderate, high), 66% of teachers indicated moderate levels, while approximately 23% of teachers indicated light intensity. Most teachers (63%) reported no barriers to incorporating physical activity into the classroom curriculum, while others (26%) reported time constraints caused by standardized testing, field trips, and substitute teachers as barriers to incorporating PAAC lessons. Very few teachers (<1%) indicated the need for additional help from PAAC staff.

Table 3 displays the results for selected items on the end-of-year teacher survey, which gathered information about the level of confidence to instruct and demonstrate physically active lessons, ratings of importance for physical activity, and levels of support received from other teachers, parents, and school administrators. Eighty teachers out of 135 (59%) completed the survey. As shown, most teachers indicated high levels of confidence to demonstrate and instruct students on how to become more physically active and incorporate physical activity into lesson plans. In addition, the majority of teachers gave very high ratings on the importance of encouraging children to become more physically active. When rating the level of support given by others for PAAC, most teachers indicated moderately high levels of support from other teachers, parents, and school administrators.

**Table 3:** Teacher ratings on selected items measuring confidence, level of importance and support for PAAC/Take 10!^® ^(N = 80)

	**Percent Occurrence**				
	
**Characteristic**	confident ↔ Extremely confident				
Demonstrate to your students how to become more physically active	1.25	2.5	25.0	38.75	32.5
Incorporate physical activity into lesson plans	2.5	7.5	21.25	46.25	22.5
Instruct students to become physically active	1.25	3.75	25.0	41.25	28.75
	
	Not at all important ↔ Extremely important				

Encourage elementary school children to become more physically active	3.75	1.25	1.25	25.0	68.75
Use healthy snacks as rewards	6.25	8.75	15.0	32.5	37.5
	
	Not at all supportive ↔ Extremely supportive				

Elementary teachers' support for PAAC	1.25	6.25	38.75	40.0	13.75
Parents' support for PAAC	10.0	13.75	41.25	22.5	12.5
School administration's support for PAAC	2.5	6.25	21.25	40.0	30.0

A summary of selected items from the end of school year evaluation completed by PAAC teachers (n = 75) are presented in Table 4. In general, most teachers reported implementing PAAC at moderately high levels by incorporating physical activity on most days, using physical activity as part of the lesson plan to break the monotony of certain subjects, and employing physically active lessons to address various learning styles. The exception was the use of the resource notebook that supplied numerous examples of active lessons for a variety of subjects, which was not used as often as expected.

**Table 4:** Ratings for Selected Characteristics of PAAC/Take 10!^® ^Lessons. Reported by Teachers (N = 75)

	Percent Occurrence				
	
Characteristic	Not at all	At least once per week	2 or more times per week	On most days	Everyday
Increased PA* by incorporating it into lesson plans	1.3	9.3	13.3	54.7	21.3
Observed children enjoying classroom activities that incorporated PA	0	4.0	13.3	50.7	32.0
Used PA as part of the lesson plan to break the monotony of certain subjects	1.3	5.3	18.7	48.0	26.7
Employed physically active lessons to address various learning styles	6.7	13.3	21.3	42.7	16.0
Enjoyed becoming physically active through implementing movement into your lesson plans	4.0	10.7	17.3	44.0	24.0
Enhanced your own sense of well-being on days that you incorporated PA into the curriculum	9.3	13.3	16.0	40.0	21.3
Used the notebook as a resource for active lessons	30.7	37.3	16.0	14.7	1.3

*PA = physical activity

### Focus group discussions

At the end of the school year, 79 teachers out of a possible 135 (~58%) participated in the focus group discussions, with approximately 13 participants in each group. Qualitative analysis of the focus group discussion transcripts revealed several common themes. Teachers reported that one of the best features of PAAC lessons are that they provide a "great teaching strategy" that helps break up the monotony of the class. Although many teachers voiced concerns at the beginning of the study about the possibility of students getting wild and out of control, teachers stated that just the opposite occurred with PAAC; it helped with behavior management, stopped the fidgeting, and made the students more alert and focused.

When asked about the worst features of PAAC, teachers indicated that they need more time to incorporate physical activity into their lesson plans, chiefly because of new requirements set forth by the No Child Left Behind Act, which holds schools accountable for academic achievement. Many teachers also specified the need to develop more lessons for students in fourth and fifth grade, stating that their students perceived the examples provided as too "babyish".

When asked about challenges and barriers to achieving 90 minutes of active lessons per week, many teachers expressed the need for lessons that could be taught within a small classroom, indicating that the current examples were designed for larger classrooms without space constraints. In addition, teachers wanted more lesson demonstrations by research assistants to gain additional ideas about how to incorporate physical activity into a regular lesson. Similarly, several teachers wanted a forum to share and learn about creative lessons that worked well in other classrooms. Several suggestions included having teachers share lessons at school staff meetings, including examples in a newsletter, or placing creative lessons developed by teachers on a website.

When we solicited information about potential benefits, both physically and academically, to students and teachers who actively participated in PAAC teachers indicated that active lessons encouraged them to be more creative, and helped students learn concepts better and improved their memorization skills. For example, one teacher stated, "it really helped my students remember things, especially spelling. Those who didn't do a PAAC activity with spelling did not do as well." With regards to physical improvements, some teachers expressed that they exercise now more than they ever have because they model the lessons with their students. Moreover, many teachers reported that the active lessons are the only physical activity the students get during most days because they stand around during recess talking with their friends, and physical education classes have been reduced to twice per week. As one teacher stated about active lessons, "This is the most I've seen some of these kids actually physically move." Others expressed that they did not think it made a difference in their bodies physically but PAAC changed their thinking about the necessity of movement within the classroom to help their students learn and retain information better.

### Principal surveys

Principals from both intervention (n = 14) and control schools (n = 10) completed surveys (22 out of 26 principals; 85% response rate although all schools were represented because some schools have divided their school population into separate buildings. For those schools, additional principals are assigned to the buildings, resulting in 2 to 3 principals for the same school. For example, one school taught kindergarten through 2^nd ^grade students in one building while 3^rd ^through 5^th ^grade students attended classes in a different building on the same grounds.

When asked how many days of physical education were offered per week, most intervention and control schools had classes 2 to 3 days per week, with the majority of classes meeting for at least 30 minutes. For one intervention school, a principal indicated that they were increasing physical activity levels by having their students prepare for the Jump Rope for Heart program, which is an American Heart Association program. Two control school principals indicated that they were increasing physical activity levels by sending home summer learning packets, which have a fitness component, and organizing active games during recess. Programs that might have increased the quality of nutrition were not installed during the school year by either intervention or control schools. Principals specified that students from both intervention and control schools were not allowed to bring soda pop in their school lunches. In summary, there were minimal competing factors or activities reported by any of the elementary school principals during year one of the study.

## Discussion

Year 1 process evaluation results suggest that the PAAC program was well accepted by intervention teachers as well as students. Direct observations and teacher self-reports indicated students enjoyed participating in active lessons and teachers enjoyed becoming physically active through implementing movement into their lesson plans. Critical to the reach and fidelity of the PAAC program, especially since curricular innovation was involved[22], was that the training sessions were well attended by teachers and other resource staff.

Despite not reaching the goal of 90 minutes per week, we believe the goal of 90 minutes per week is achievable. As explained by Locke and Latham,[23] the concept of self-efficacy is important in goal-setting theory and is consistent with social cognitive theories. Goal-setting theorists explain that individuals with high self-efficacy set higher goals than do people with low self-efficacy. These individuals are also more committed to assigned goals, and will find and use better strategies to attain those goals. According to Bandura's theory [24], behavioral changes are mostly mediated by self-efficacy–the belief that one can successfully perform a desired behavior. He explains that self-efficacy expectations determine how much effort people will give and how long they will continue to try to meet a goal even if obstacles exist. Similarly, although PAAC teachers met with barriers when incorporating physically active lessons, they reported high self-efficacy to perform physically active lessons, and indicated that they were confident about their ability to incorporate PA into lesson plans and to instruct students to become more physically active.

Not only is the belief that an individual can attain a specific goal a significant factor in goal-setting theory but another key moderator is the importance placed on the outcomes expected as a result of working to attain a goal [23]. PAAC teachers gave very high ratings on the importance of encouraging children to become more active, and indicated that they were committed to helping achieve higher rates of PA. Moreover, both teachers and students enjoyed the active lessons compared to the traditional ones. Because there is consistent research evidence that enjoyment is associated with participation in physical activity among children [25], we believe teachers will continue to employ physically active lessons within their classrooms.

For the next school year, information gained on program fidelity will be used to refine protocols and program materials in order to support teachers in the continual use of PAAC activities, and to provide strategies to achieve the targeted goal of 90 minutes of PAAC lessons per week.

### Strengths

One of the strengths of the process evaluation is that both quantitative and qualitative methods were used to allow for a more thorough examination of program fidelity and implementation issues. Quantitative data regarding minutes of physical activity and number of lessons taught were collected weekly, which helped to determine if teachers were meeting the minimum prescription for the intervention. Weekly reporting also identified struggling teachers without delay. In this manner, assistance could be provided before excessive time elapsed. Data were also collected to help determine challenges teachers faced implementing the intervention, allowing study personnel to help minimize barriers to increasing physical activity within their curriculum. Areas of help provided by PAAC staff included lesson development, time management (e.g., determined how to include PAAC lessons within the framework of their lesson plans) and classroom management (e.g., providing cool down tips in order to help keep students focused after a physically active lesson). Through information gained by focus group discussions, we were able to modify the protocol to address implementation issues conveyed by teachers. For example, for the new academic year, teachers were supplied with several examples of lessons for fourth and fifth grade students designed to be more age appropriate. In addition, we solicited from teachers a number of creative lessons that students found enjoyable and were easily implemented. These particular lessons were placed on the PAAC website, allowing other teachers to gain examples of successful lessons.

Another strength of the evaluation was the collection of key process variables in both intervention and control schools. Although research staff costs, the burden of collecting and managing large amounts of data, and the complexity of school schedules were important considerations, tracking key process variables (i.e., physical activity levels, student enjoyment, and possible contamination factors) in both intervention and control schools provided essential information on fidelity, program context, and possible threats to the internal validity of the PAAC program.

### Limitations

One of the limitations of the process evaluation data was that it relied upon teachers to self-report weekly data on how much and into what particular subjects was physical activity incorporated into lessons. Although the majority of teachers reported information in a timely manner, several teachers delayed reporting until being reminded by program staff, which could have potentially introduced recall bias. Another limitation was the low response rate for the end-of-year surveys. To ensure a greater response for the next school year, we have planned to administer the survey a week earlier to teachers so it does not interfere with end-of-school year activities and to reduce respondent burden during this particularly busy time of year.

### Practice implications

One of the goals of the PAAC project was to develop a minimal intervention that increased physical activity during school without reducing time allocated for classroom instruction, given the pressure to meet the No Child Left Behind Act's accountability provisions, in which schools must close the achievement gap, making sure all students achieve academic proficiency, Results from the process evaluation demonstrated that it is possible to incorporate physical activity within the curriculum by modifying existing academic lessons, thereby not competing for instructional time.

One of the major advantages of the PAAC project is that it is a minimal intervention that can be easily disseminated, requiring no change to the current curriculum, few additional supplies (if any) and minimal cost to schools. Some of the reasons for the success of PAAC during Year 1 can be attributed to the training provided to teachers and both the teachers' and students' positive responses to the physically active lessons. Further, teachers were given much flexibility and choice on how to integrate the intervention within their classroom (e.g., in what academic subjects, and how many times per day).

## List of abbreviations

PAAC: Physical activity across the curriculum; ILSI CHP: International life sciences institute center for health promotion; SOFIT: System for observing fitness instruction time; CATCH: Child and adolescent trial for cardiovascular health.

## Competing interests

The authors declare that they have no competing interests.

## Authors' contributions

JD conceived of the study. CG, BS, KD, JG, JR, RW, KS, DS, MM, and JD helped to carry out the PAAC study, participated in the design and coordination, and helped to draft the manuscript. In addition, MM and KS performed the statistical analyses. SW and BB helped to draft the manuscript. All authors read and approved the final manuscript.

## References

[B1] Strauss RS, Pollack HA (2001). Epidemic increase in childhood overweight, 1986-1998. JAMA.

[B2] Hedley AA, Ogden CL, Johnson CL, Carroll MD, Curtin LR, Flegal KM (2004). Prevalence of overweight and obesity among US children, adolescents, and adults, 1999-2002.[see comment]. JAMA.

[B3] DHHS (2003). Partners invited to participate in Steps to a HealthierUS.. Federal Register.

[B4] Snyder T, Hoffman C (2002). Digest of Education Statistics 2001.

[B5] Kann L, Brener ND, Allensworth DD (2001). Health education: results from the School Health Policies and Programs Study 2000. Journal of School Health.

[B6] Wechsler H, Devereaux RS, Davis M, Collins J (2000). Using the school environment to promote physical activity and healthy eating. Preventive Medicine.

[B7] Epstein LH, McCurley J, Wing RR, Valoski A (1990). Five-year follow-up of family-based behavioral treatments for childhood obesity. J Consult Clin Psych.

[B8] Gortmaker S, Peterson K, Wiecha J, Sobol AM, Dixit S, Fox MK, Laird N (1999). Reducing obesity via a school-based interdisciplinary intervention among youth. Planet Health. Archives of Pediatrics & Adolescent Medicine.

[B9] Rossi PH, Freeman HE (1985). Evaluation: a systematic approach.

[B10] Rossi PH (1978). Issues in the evaluation of human services delivery. Evaluation Quarterly.

[B11] Linnan L, Steckler A, Steckler ALL (2002). Process evaluation and public health interventions: an overview.. Process evaluation in public health interventions and research: theory and practice.

[B12] Baranowski T, Stables G (2000). Process evaluation of the 5-a-Day projects. Health Educ Behav.

[B13] Glasgow RE, Vogt TM, Boles SM (1999). Evaluating the public health impact of health promotion interventions: the RE-AIM Framework. Am J Public Health.

[B14] McKenzie TL, Sallis JF, Nader PR (1991). SOFIT: System for observing fitness instruction time. J Teaching Phys Educ.

[B15] McGraw SA, Stone EJ, Osganian SK, Elder JP, Perry CL, Johnson CC, Parcel GS, Webber LS, Luepker RV (1994). Design of process evaluation within the Child and Adolescent Trial for Cardiovascular Health (CATCH). Health Education Quarterly Suppl.

[B16] Elder JP, McGraw SA, Stone EJ, Reed DB, Harsha DW, Greene T, Wambsgans KC (1994). CATCH: process evaluation of environmental factors and programs. Health Education Quarterly Suppl.

[B17] Perry CL, Stone EJ, Parcel GS, Ellison RC, Nader PR, Webber LS, Luepker RV (1990). School-based cardiovascular health promotion: the child and adolescent trial for cardiovascular health (CATCH). Journal of School Health.

[B18] McKenzie TL, Strikmiller PK, Stone EJ, Woods SE, Ehlinger SS, Romero KA, Budman ST (1994). CATCH: physical activity process evaluation in a multicenter trial. Health Education Quarterly Suppl.

[B19] Krueger RA, Morgan DLKRA (1998). Moderating focus groups. The focus group kit.

[B20] Miles MB, Huberman AM (1984). Qualitative data analysis: a sourcebook of new methods.

[B21] Cook TD, Campbell DT (1979). Quasi-experimentation. Design and analysis issues for field settings.

[B22] Story M, Mays RW, Bishop DB, Perry CL, Taylor G, Smyth M, Gray C (2000). 5-a-Day Power Plus: process evaluation of a multicomponent elementary school program to increase fruit and vegetable consumption. Health Education & Behavior.

[B23] Locke EA, Latham GP (1990). A theory of goal setting and task performance..

[B24] Bandura A (1977). Self-efficacy: toward a unifying theory of behavior change. Psych Rev.

[B25] Sallis JF, Prochaska JO, Taylor WC (2000). A review of correlates of physical activity of children and adolescents. Med Sci Sports Exer.

